# Electrochemical and chemical cycle for high-efficiency decoupled water splitting in a near-neutral electrolyte

**DOI:** 10.1038/s41563-023-01767-y

**Published:** 2024-01-09

**Authors:** Ilya Slobodkin, Elena Davydova, Matan Sananis, Anna Breytus, Avner Rothschild

**Affiliations:** 1 Department of Materials Science and Engineering, Technion – Israel Institute of Technology, Haifa, Israel; 2 The Nancy and Stephen Grand Technion Energy Program (GTEP), Technion – Israel Institute of Technology, Haifa, Israel; 3 The Stewart and Lynda Resnick Sustainability Center for Catalysis (RSCC), Technion – Israel Institute of Technology, Haifa, Israel

**Keywords:** Electrocatalysis, Electrocatalysis

## Abstract

Green hydrogen produced by water splitting using renewable electricity is essential to achieve net-zero carbon emissions. Present water electrolysis technologies are uncompetitive with low-cost grey hydrogen produced from fossil fuels, limiting their scale-up potential. Disruptive processes that decouple the hydrogen and oxygen evolution reactions and produce them in separate cells or different stages emerge as a prospective route to reduce system cost by enabling operation without expensive membranes and sealing components. Some of these processes divide the hydrogen or oxygen evolution reactions into electrochemical and chemical sub-reactions, enabling them to achieve high efficiency. However, high efficiency has been demonstrated only in a batch process with thermal swings that present operational challenges. This work introduces a breakthrough process that produces hydrogen and oxygen in separate cells and supports continuous operation in a membraneless system. We demonstrate high faradaic and electrolytic efficiency and high rate operation in a near-neutral electrolyte of NaBr in water, whereby bromide is electro-oxidized to bromate concurrent with hydrogen evolution in one cell, and bromate is chemically reduced to bromide in a catalytic reaction that evolves oxygen in another cell. This process may lead the way to high-efficiency membraneless water electrolysis that overcomes the limitations of century-old membrane electrolysis.

## Abstract (web summary)

Hydrogen produced by water splitting using renewable electricity is key to achieve net-zero carbon emissions. Decoupling hydrogen and oxygen evolution reactions during electrolysis is attractive but efficiency and operational challenges remain. A process producing hydrogen and oxygen in separate cells and supporting continuous operation in a membraneless system is now proposed.

## Main

Green hydrogen produced by water splitting using renewable energies is essential to reduce greenhouse gas emissions, especially in hard-to-abate industrial sectors such as steel, cement and ammonia production. At present, water electrolysis technologies are uncompetitive with the low-cost production of grey hydrogen by steam methane reforming^1^. Therefore, there is a pressing need to improve water electrolysis to support low-cost production of green hydrogen at the terawatt scale. Towards this aim, decoupled water electrolysis (DWE), wherein the hydrogen and oxygen evolution reactions (HER and OER, respectively) are decoupled in time and/or place, has emerged as a disruptive concept that has spurred innovative efforts to overcome the limitations of water electrolysis^2–4^. DWE may lead the way to safe operation without membranes^5–8^, providing new opportunities to reshape water electrolysis and potentially overcome the fundamental barriers of this century-old technology.

DWE was first reported by Symes and Cronin in 2013, introducing phosphomolybdic acid as a soluble redox couple (SRC) that functions as an electron-coupled-proton buffer and mediates the electron-coupled-proton exchange between the anodic OER and cathodic HER^9^. Despite the process generating oxygen and hydrogen at different times in stepwise stages, a membrane was used to prevent redox shuttling of the polyoxomolybdate anions between the electrodes, and the efficiency was lower than that of conventional water electrolysis. Low efficiency is an inherent disadvantage of this approach since the oxidation and reduction overpotentials of the SRC add up to those of the OER and HER, thus necessitating a larger voltage than in conventional water electrolysis^4^. Subsequent studies pursuing this approach introduced different electron-coupled-proton buffers in acidic electrolytes, but the efficiency remained low and a membrane was still necessary^10–14^.

Another DWE scheme was reported by Chen et al.^5^ and by Landman et al.^6^, replacing the SRC by solid redox electrodes (SRE). To this end, nickel (oxy)hydroxide electrodes such as those commonly used in rechargeable alkaline batteries were employed as auxiliary electrodes that mediate the hydroxide ion (OH^–^) exchange between the HER at the cathode of one cell and the OER at the anode of another cell. Thereby the electrolytic cell was divided into two separate cells that generate hydrogen and oxygen remotely from each other, paving the way for membraneless DWE. This approach requires batch operation to regenerate the auxiliary electrodes, whereas SRCs support continuous operation much like conventional electrolysers^15^.

A different approach was reported by Rausch et al., introducing an electrochemical and chemical cycle whereby silicotungstic acid was reduced electrochemically at the cathode while oxygen evolved at the anode with an electrolytic efficiency of 63%_HHV_ (based on the higher heating value of hydrogen), and then transferred into another cell where it was oxidized chemically and released hydrogen upon contact with a platinum catalyst^16^. The next leap was reported by Dotan et al., introducing an electrochemical and thermally activated chemical (ETAC) cycle that divides the OER into two sub-reactions and enables operation in near thermoneutral conditions^7^. This was achieved by cycling a nickel hydroxide anode between an electrochemical stage (E) that charges the anode to nickel oxyhydroxide while hydrogen evolves at the cathode, and a thermally activated chemical stage (TAC) that reduces it back to nickel hydroxide and evolves oxygen without applying electricity.

The ETAC process enables membraneless operation with a remarkable electrolytic efficiency of 98.7%_HHV_ (at the cell level) at a current density of 50 mA cm^–2^ (ref. ^7^). But it also presents new challenges that emerge from batch operation and swinging between cold and hot electrolytes in the E and TAC stages, as well as from capacity and rate limitations of the nickel (oxy)hydroxide anodes. These limitations can be circumvented altogether by shifting the charge storage from the solid nickel (oxy)hydroxide anode to the liquid electrolyte, thereby enabling continuous instead of batch operation and avoiding kinetic limitations that arise from solid-state diffusion and phase transformations in the SRE^17^. This work presents a proof of concept of this new approach, using a SRC that stores and releases oxygen instead of hydrogen^16^, and demonstrates membraneless DWE in a continuous and isothermal process (without thermal swings) with high efficiency and high current density.

## Concept

We propose an electrochemical and chemical cycle that divides the OER into two sub-reactions, electrochemical and chemical, similarly to the ETAC cycle^7^. But instead of using a nickel (oxy)hydroxide anode, we propose a SRC that supports continuous operation and an isothermal process with high efficiency and at a high rate. The reduced SRC (red) is oxidized in an electrochemical reaction (red → ox + *n*e^–^, where *n* is the number of electrons (e^–^)) that complements the HER without evolving oxygen or other volatile side products; and, in the presence of a suitable catalyst, the oxidized SRC (ox) evolves oxygen spontaneously in a chemical reaction that reduces it back to its reduced state (ox → red + O_2_). To provide a driving force for this chemical reaction, the SRC should have a reversible redox potential (*E*^0^) above the thermodynamic OER potential (1.23 V_RHE_, with respect to the reversible hydrogen electrode), whereas for high efficiency it should be oxidized at a low overpotential and ideally below the OER onset potential (∼1.6 V_RHE_ for state-of-the-art OER catalysts)^18^. This dictates a reversible redox potential of ∼1.4 V_RHE_, similar to that of nickel (oxy)hydroxide^7^. Based on these criteria, the bromide (Br^–^) / bromate (${{\rm{BrO}}}_{3}^{-}$) couple (*E*^0^ = 1.42 V_RHE_)^19^ was selected. The salts of both the reduced and oxidized species, NaBr and NaBrO_3_, have high solubility in water, 946 and 394 g l^–1^, respectively (at 25 °C)^20^. Moreover, the bromine (Br_2_) produced at the anode is denser than water (3.1 g cm^–3^)^20^ and is highly soluble in water (34 g l^–1^ at 25 °C)^21^, which minimizes the risk of evaporation and entrainment by the hydrogen bubbles produced at the cathode. This makes the ${{\rm{Br}}}^{-}/{{\rm{BrO}}}_{3}^{-}$ couple preferable over the ${{\rm{Cl}}}^{-}/{{\rm{ClO}}}_{3}^{-}$ couple that produces volatile chlorine (Cl_2_) with low solubility in water (6.3 mg l^–1^ at 25 °C)^22^, which results in a faradaic loss^23^.

**Fig. 1:**
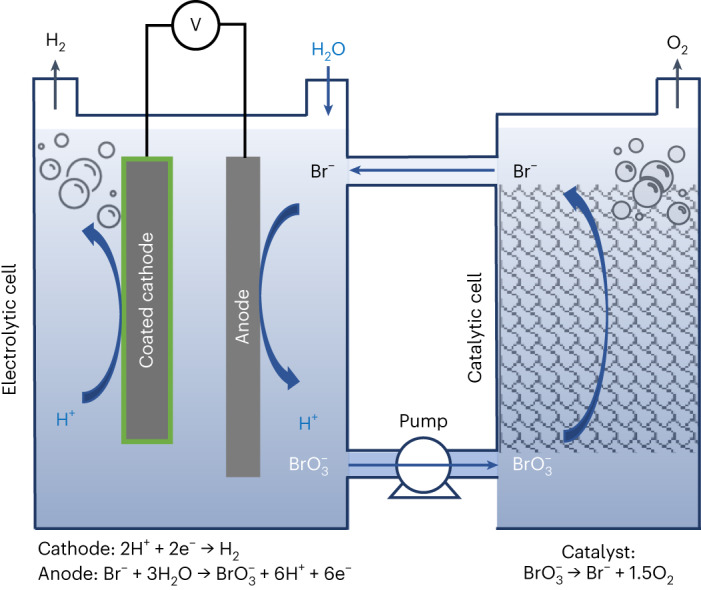
Proposed DWE process. Schematic illustration of the proposed DWE process with continuous generation of H_2_ and O_2_ in separate electrolytic and catalytic cells using Br^–^/BrO_3_^–^ as a soluble redox couple. The oxidized bromate-rich (BrO_3_^–^) electrolyte flows from the bottom of the electrolytic cell to the bottom of the catalytic cell, where it is reduced to bromide (Br^–^) by a catalyst and flows from the top of the catalytic cell back to the top of the electrolytic cell. The green coating represents a semipermeable chromium hydroxide layer.

Figure 1 illustrates our DWE process for the decoupled production of hydrogen and oxygen in separate cells, using ${{\rm{Br}}}^{-}/{{\rm{BrO}}}_{3}^{-}$ as a SRC that stores and releases oxygen by turns. The electrolytic cell comprises a cathode that generates hydrogen by the HER (reaction 1) and an anode where the bromide electro-oxidation reaction (reaction 2) takes place:

1 $$2{{\rm{H}}}^{+}+2{{\rm{e}}}^{-}\to {{\rm{H}}}_{2}$$

2 $$2{\rm{B}}{{\rm{r}}}^{-}\to {\rm{B}}{{\rm{r}}}_{2}+2{{\rm{e}}}^{-}$$

According to studies on bromide electrolysis^24,25^, the bromine molecules (Br_2_) produced at the anode react with water in the bulk of the electrolyte to form hypobromous acid (HBrO, reaction 3) that forms hypobromite anions (BrO^–^) and protons by dissociation (reaction 4). The hypobromite anions react with hypobromous acid to form bromate anions (${{\rm{BrO}}}_{3}^{-}$, reaction 5), the desired product:

3 $${\rm{B}}{{\rm{r}}}_{2}+{{\rm{H}}}_{2}{\rm{O}}\rightleftharpoons {\rm{HBrO}}+{{\rm{H}}}^{+}+{\rm{B}}{{\rm{r}}}^{-}$$

4 $${\rm{HBrO}} \ {\rightleftharpoons {\rm{H}}}^{+}+{\rm{Br}}{{\rm{O}}}^{-}$$

5 $$2{\rm{HBrO}}+{\rm{Br}}{{\rm{O}}}^{-}\to {\rm{Br}}{{\rm{O}}}_{3}^{-}+2{\rm{B}}{{\rm{r}}}^{-}+2{{\rm{H}}}^{+}$$

The overall anode-related process, reactions 2–5, can be summarized by reaction 6:

6 $${\rm{B}}{{\rm{r}}}^{-}+3{{\rm{H}}}_{2}{\rm{O}}\to {\rm{Br}}{{\rm{O}}}_{3}^{-}+6{{\rm{H}}}^{+}+6{{\rm{e}}}^{-}$$

resulting in an ${{\rm{e}}}^{-}/{{\rm{H}}}_{2}/{{\rm{BrO}}}_{3}^{-}$ ratio of 6:3:1. For brevity, Fig. 1 illustrates the anodic-related reactions (reactions 2–5) as one (reaction 6).

The HBrO and BrO^–^ intermediate products may lead to undesired side reactions^25^:

7 $$6{\rm{Br}}{{\rm{O}}}^{-}+3{{\rm{H}}}_{2}{\rm{O}}\to 2{\rm{Br}}{{\rm{O}}}_{3}^{-}+6{{\rm{H}}}^{+}+4{\rm{B}}{{\rm{r}}}^{-}+1.5{{\rm{O}}}_{2}+6{{\rm{e}}}^{-}$$

8 $${\rm{HBrO}}+2{{\rm{e}}}^{-}\to {\rm{B}}{{\rm{r}}}^{-}+{\rm{O}}{{\rm{H}}}^{-}$$

9 $${\rm{Br}}{{\rm{O}}}^{-}+{{\rm{H}}}_{2}{\rm{O}}+2{{\rm{e}}}^{-}\to {\rm{B}}{{\rm{r}}}^{-}+2{\rm{O}}{{\rm{H}}}^{-}$$

10 $${\rm{Br}}{{\rm{O}}}_{3}^{-}+{3{\rm{H}}}_{2}{\rm{O}}+6{{\rm{e}}}^{-}\to {\rm{B}}{{\rm{r}}}^{-}+6{\rm{O}}{{\rm{H}}}^{-}$$

11 $${\rm{Br}}{{\rm{O}}}_{3}^{-}+{2{\rm{H}}}_{2}{\rm{O}}+4{{\rm{e}}}^{-}\to {\rm{B}}{{\rm{rO}}}^{-}+4{\rm{O}}{{\rm{H}}}^{-}.$$

Operation at 60 °C and pH 8 was found to provide optimal conditions to suppress oxygen evolution (reaction 7) and achieve close to 100% faradaic efficiency for bromate production (reaction 6)^26,27^. To suppress the cathodic backward reactions (reactions 8–11), a small amount (1–3 g l^–1^) of sodium dichromate (Na_2_Cr_2_O_7_) is added to the sodium bromide (NaBr) aqueous electrolyte. The dichromate anions (Cr_2_O_7_^2–^) are reduced and deposited on the cathode, coating it with a semipermeable chromium hydroxide layer (illustrated by the green layer in Fig. 1) that suppresses the cathodic loss reactions while allowing the HER to occur without hindrance. This enables us to achieve a high faradaic efficiency without needing a membrane to divide the cell into anodic and cathodic compartments.

The catalytic cell comprises a column embedded with a catalyst (Fig. 1, right) that facilitates the catalytic decomposition of bromate anions (${{\rm{BrO}}}_{3}^{-}$) into bromide (Br^–^) and oxygen:

12 $${2{\rm{BrO}}}_{3}^{-}\to {2{\rm{Br}}}^{-}+{3{\rm{O}}}_{2}.$$

The two cells are connected into a flow system that provides continuous electrolyte flow from one cell to another, as illustrated in Fig. 1.

## Proof of concept

The feasibility of the proposed DWE process was validated separately in two sets of experiments that examine the performance of the electrolytic and catalytic subprocesses. Then, complementary measurements were carried out combining the two subprocesses into a unified batch-to-bath process that splits water into hydrogen and oxygen in separate cells.

### Electrolytic process

**Fig. 2:**
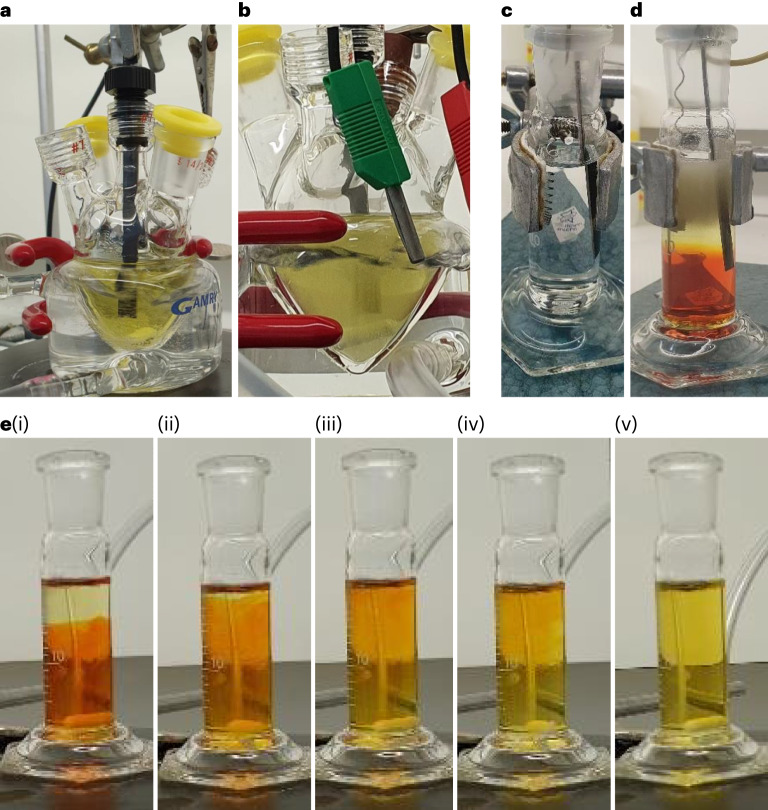
Bromide electrolysis tests. **a**–**e**, Photographs of the electrolytic cells we used to examine the bromide electrolysis process with stirred (**a** and **b**) and still (**c**–**e**) electrolytes. Panels **a** and **c** are before electrolysis; **b** and **d** are during electrolysis; and **e**(i)–(v) shows sequential snapshots during subsequent stirring after electrolysis (Supplementary Video 2). The electrolyte composition and other experimental conditions are summarized in Table 1.

The electrolytic process was examined in two operational modes. The first (main) mode corresponds to bromate electrolysis in 1.5 M NaBr aqueous electrolyte with the addition of 3.8 mM Na_2_Cr_2_O_7_, where the electrolyte was heated to 60 °C and stirred during the process. In the second mode, we examined the possibility of carrying out the process without the toxic Na_2_Cr_2_O_7_ additive by using the phase separation between the high-density bromine (Br_2_) that forms on the anode (reaction 2) and the rest of the electrolyte to minimize the diffusion of reaction products to the cathode. In the second mode, the electrolysis was carried out in an unheated electrolyte (at room temperature) without stirring. Figure 2 presents photographs of the electrolysis tests carried out in the first and second modes (Fig. 2a,b and Fig. 2c,d, respectively; the electrolyte composition and other experimental conditions are summarized in Table 1). In both cases, the anode and cathode were placed in an electrolytic cell with no membrane or diaphragm division. The addition of Na_2_Cr_2_O_7_ in the first operational mode resulted in a yellowish solution (prior to electrolysis), as shown in Fig. 2a, whereas in its absence in the second mode the electrolyte was colourless (Fig. 2c). During operation in the first mode, the whole volume of the electrolyte becomes cloudy (Fig. 2b) due to the evolution of hydrogen bubbles that were stirred throughout the cell. The electrolyte colour remained yellowish, comprising contributions from both the Cr_2_O_7_^2−^ anions and the bromide oxidation intermediates. The operation in the second mode resulted in intense hydrogen bubble formation at the top of the cell, along with a phase separation between the red Br_2_-rich oxidized solution that sank down to the bottom of the cell, and the yellowish solution that contained oxidized bromine species in the upper part of the cell (Supplementary Video 1). Subsequent stirring turned the phase-separated red and yellowish solutions into a homogeneous yellowish solution (Fig. 2e(i)–(v) and Supplementary Video 2), indicating that the Br_2_-rich solution reacted with the rest of the electrolyte according to reactions 3–5. However, a residual amount of unreacted intermediate products remained, as indicated by the yellow colour of the stirred solution.

The faradaic efficiency of bromide electro-oxidation was examined for both operational modes, with and without Na_2_Cr_2_O_7_, by electrolysing 20 ml of 1.5 M NaBr electrolyte (without buffers) for 5.36 hours at a current of 600 mA (total charge, 11,578 Coulomb) and analysing the resulting bromate content by iodometric titration (Supplementary Video 3). Considering that six electrons are needed to oxidize a bromide anion to a bromate anion (reaction 6), this charge should produce 0.02 moles of bromate anions at 100% faradaic efficiency and convert 2/3 (1 M) of the bromide anions in the initial electrolyte (20 ml of 1.5 M NaBr) to bromate anions. In the first operational mode (Fig. 2b), the faradaic efficiency was 98 ± 2%, indicating successful suppression of the cathodic side reactions (reactions 8–11) as well as the anodic OER (reaction 7). A direct confirmation of no OER interference is presented in Supplementary Video 4, showing bromide electrolysis in a Hoffman apparatus with no oxygen evolution. Without the Na_2_Cr_2_O_7_ additive (but otherwise the same conditions), the faradaic efficiency dropped to 10 ± 1%, indicating the important role of Na_2_Cr_2_O_7_ to prevent reactions 8–11 by forming a polyoxide cathodic barrier, as reported elsewhere^24,26–31^.

**Extended Data Fig. 1:**
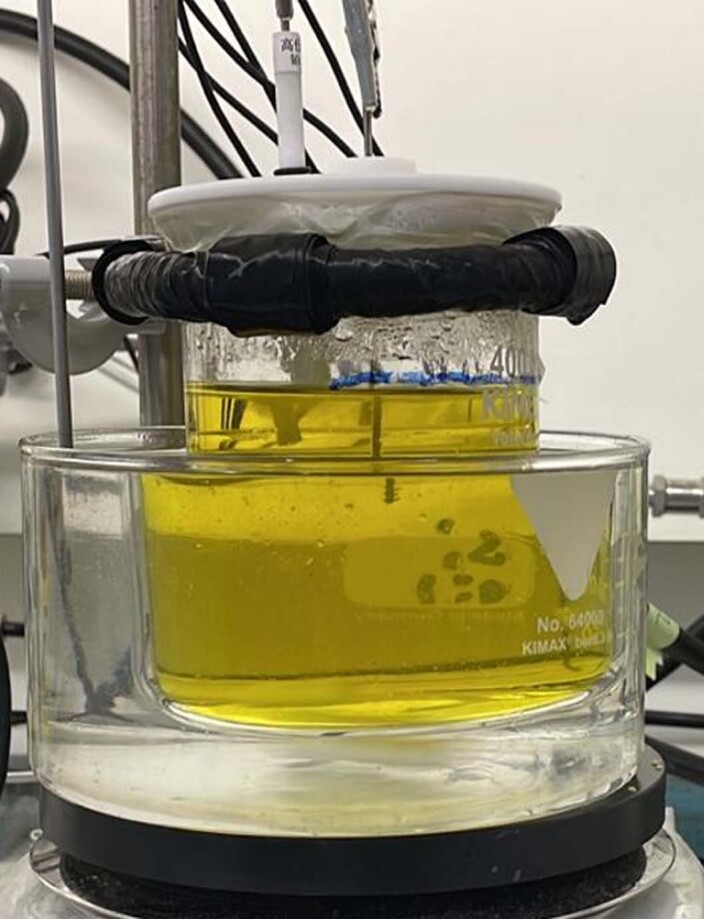
Long term electrolysis stability. A photograph of the measurement setup.

**Extended Data Fig. 2:**
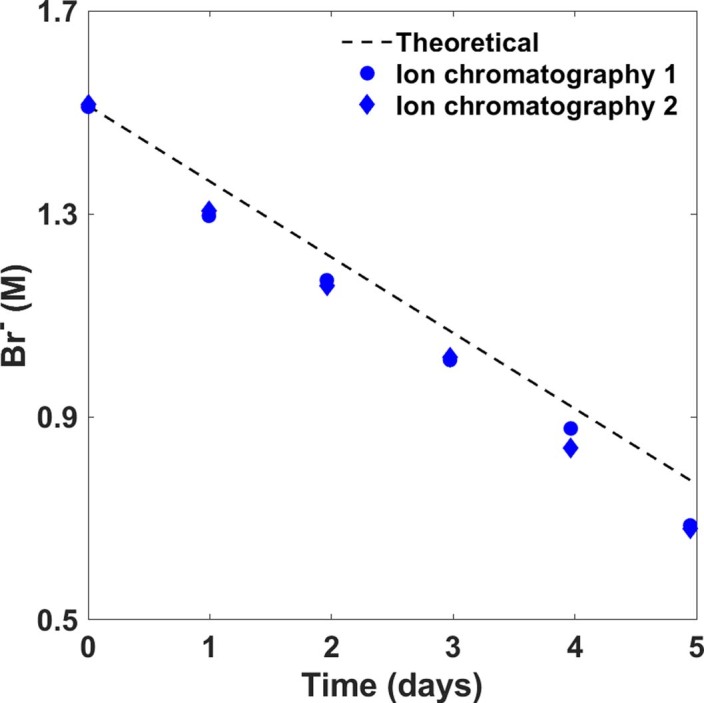
Long term electrolysis stability. Results showing the **bromide (Br**^**–**^**)** concentration during five days of continuous electrolysis of 300 ml aqueous solution of 1.5 M NaBr (initial composition) with 0.3 M borate buffer and 3.8 mM Na_2_Cr_2_O_7_, at a current of 300 mA, temperature of 60 °C, and stirring rate of 400 rpm. The data points present ion chromatography measurements of two duplicate samples.

**Extended Data Fig. 3:**
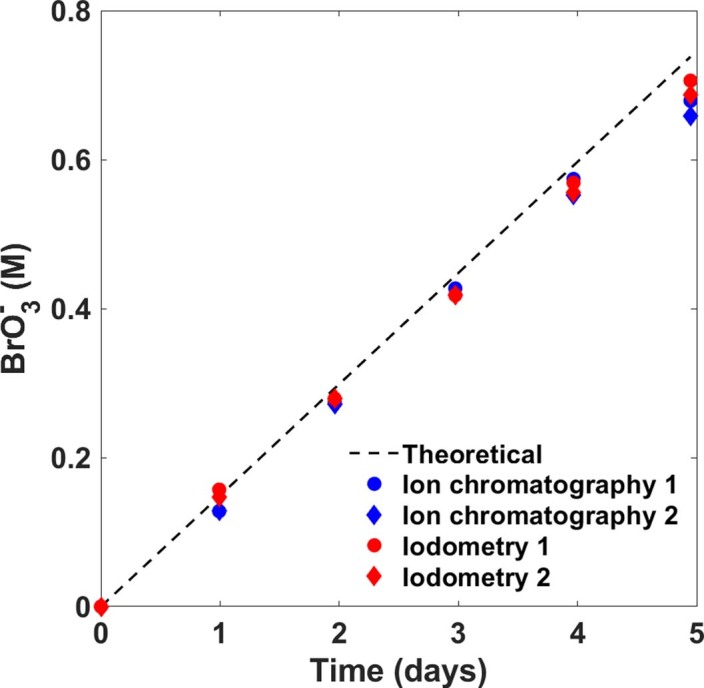
Long term electrolysis stability. Results showing the **bromate (BrO**_**3**_^**–**^**)** concentration during five days of continuous electrolysis of 300 ml aqueous solution of 1.5 M NaBr (initial composition) with 0.3 M borate buffer and 3.8 mM Na_2_Cr_2_O_7_, at a current of 300 mA, temperature of 60 °C, and stirring rate of 400 rpm. The data points present ion chromatography (blue) and iodometric titration (red) of two duplicate samples.

In the second operational mode (Fig. 2d), without Na_2_Cr_2_O_7_, the faradaic efficiency was 72 ± 2% without stirring, and it dropped to 13 ± 1% with stirring, demonstrating the effectiveness of the spontaneous phase separation between the oxidized electrolyte and the rest of the electrolyte in suppressing the cathodic loss reactions (reactions 8–11). The faradaic efficiency may be further enhanced by removing the oxidized electrolyte from the bottom of the cell, as illustrated in Fig. 1. This approach may lead the way to high-efficiency operation in a benign NaBr electrolyte without Na_2_Cr_2_O_7_. A similar approach has been reported in membraneless zinc–bromine redox flow batteries, harnessing the phase separation in the electrolyte to suppress backward reactions like those occurring in our system^32^. An alternative approach to adding Na_2_Cr_2_O_7_ to the electrolyte is precoating the cathode (ex situ) with a chromium polyoxide (or other) layer instead of in situ deposition of Cr_2_O_7_^2–^ anions during operation in the presence of Na_2_Cr_2_O_7_. We have achieved partial success pursuing this approach by using a precoated cathode that was installed after going through previous electrolysis tests with Na_2_Cr_2_O_7_ in the electrolyte, reaching a faradaic efficiency of 80 ± 2% in subsequent tests without Na_2_Cr_2_O_7_. We suspect that the lower faradaic efficiency of the precoated cathode with respect to in situ coating during electrolysis in the presence of Na_2_Cr_2_O_7_ in the electrolyte may be ascribed to the detachment of small segments of the coating layer during operation. In the presence of Cr_2_O_7_^2–^ anions in the electrolyte, the barrier layer is more effective than the ex situ precoating, probably due to self-healing of the polyoxide layer during operation. In addition, Na_2_Cr_2_O_7_ also serves as a buffer and without it the pH shifts to high values (Table 1) that may promote parasitic oxygen evolution (reaction 7). Long-term tests demonstrate the process stability in the presence of Na_2_Cr_2_O_7_ during continuous operation over five days, as shown in Extended Data Fig. 1, by monitoring the concentration of bromide and bromate in the electrolyte (Extended Data Figs. 2 and 3, respectively).

**Fig. 3:**
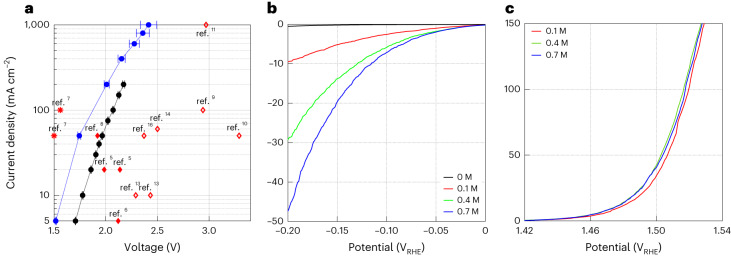
Electrolytic efficiency of bromide electrolysis. **a**, Steady-state current density versus voltage (*IR* corrected) results obtained by galvanostatic measurements at different current densities during bromide electrolysis with (blue ● symbols) and without (black ● symbols) borate buffer (0.7 M). For a comparison, results from previous DWE reports are marked by red symbols (◊ for SRC, ♦ for SRE and * for ETAC) and labelled by their respective reference numbers. The electrolyte composition at the beginning of the electrolysis measurements was 1.5 M NaBr with 3.8 mM Na_2_Cr_2_O_7_, with and without borate buffer. The electrolyte was heated to 60 °C and stirred at 400 r.p.m. **b**,**c**, Current density versus potential curves corrected) of the Pt foil cathode and RuO_2_–TiO_2_/Ti DSA (anode), respectively, obtained by LSV measurements with a potential scan rate of 1 mV s^–1^. The experimental conditions of all the measurements are summarized in Table 2. The data points and error bars in **a** present the mean values and standard deviation of the galvanostatic measurements presented in Extended Data Fig. 5 (the numerical values are presented in Supplementary Table 1b).

**Extended Data Fig. 4:**
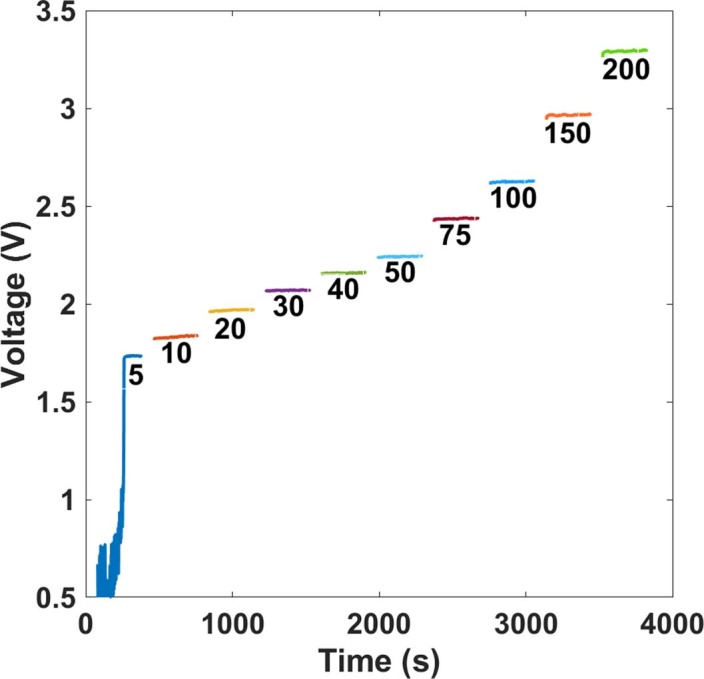
Chronopotentiometry measurements at different current densities in NaBr electrolyte without a buffer. Electrolyte composition: 1.5 M NaBr with 3.8 mM Na_2_Cr_2_O_7_. The electrolyte was heated to 60 °C and stirred at a rate of 400 rpm. The current densities are indicated by the labels in the figures (in units of mA/cm^2^). The voltage is presented without *IR* correction.

**Extended Data Fig. 5:**
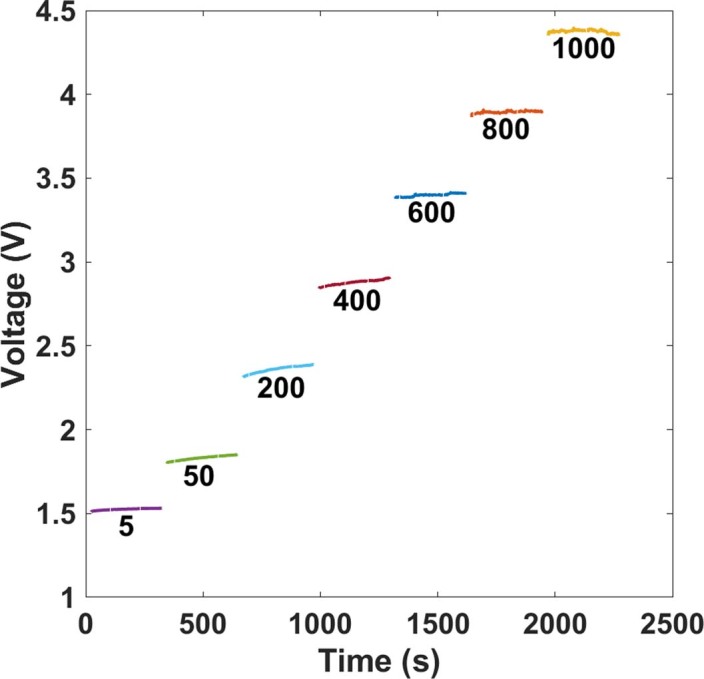
Chronopotentiometry measurements at different current densities in NaBr electrolyte with a borate buffer. Electrolyte composition: 1.5 M NaBr, 0.7 M borate buffer, 3.8 mM Na_2_Cr_2_O_7_. The electrolyte was heated to 60 °C and stirred at a rate of 400 rpm. The current densities are indicated by the labels in the figures (in units of mA/cm^2^). The voltage is presented without *IR* correction.

**Extended Data Fig. 6:**
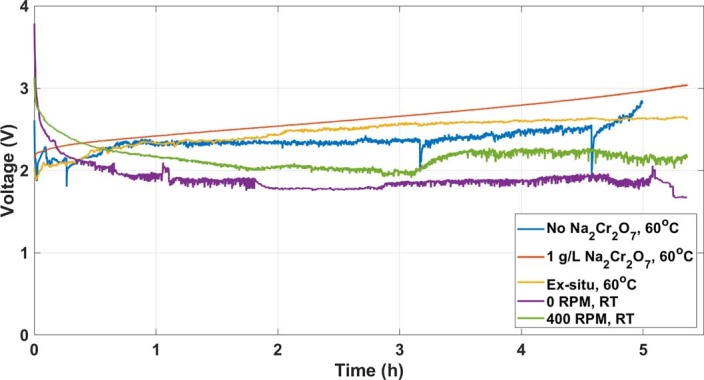
Bromide electrolysis stability. The voltage (*IR* corrected) as a function of time during prolonged galvanostatic measurements at a current of 600 mA in 1.5 M NaBr electrolyte with (red curve) or without 3.8 mM Na_2_Cr_2_O_7_ (all other curves) measured at 60 °C (blue, red and orange curves) or at room temperature (purple and green curves) with (all curves expect for the purple curve) or without stirring (purple curve). Pristine Pt foil or coil (at 60^o^C or room temperature, RT, respectively) cathode and RuO_2_/TiO_2_ DSA anode were used in the measurements, except for the one presented by the orange curve where a pre-coated Pt foil cathode from previous electrolysis tests was used. The voltage drift in the red curve results from changes in the electrolyte composition due to conversion of bromide to bromate during the measurement.

In addition to the faradaic efficiency measurements, the electrolytic (that is, voltage) efficiency was measured for the first operational mode (Fig. 2b), which demonstrated the highest faradaic efficiency (98 ± 2%). This was done by two-electrode galvanostatic voltammetry measurements at different current densities ranging from 5 to 1,000 mA cm^–2^ without and with borate buffer (Extended Data Figs. 4 and 5, respectively). Figure 3a presents the steady-state current density versus voltage results (*IR* corrected, where *I* is the current and *R* the series resistance) obtained for bromide electrolysis in unbuffered (black) and buffered (0.7 M borate buffer, blue) 1.5 M NaBr electrolytes with 3.8 mM Na_2_Cr_2_O_7_, heated to 60 °C and stirred at 400 r.p.m.; the experimental conditions of these measurements are summarized in Table 2. Introducing 0.7 M borate buffer decreases the cell voltage by ∼0.2 V at current densities up to 50 mA cm^–2^, resulting in a low onset voltage of 1.5 V at 5 mA cm^–2^. The reduction in cell voltage remains notable even at 200 mA cm^–2^. A cell voltage of 2.4 V was obtained at a high current density of 1 A cm^–2^. Consequently, the electrolytic cell efficiency increased from 86 to 97%_HHV_ at 5 mA cm^–2^, and from 75 to 85%_HHV_ at 50 mA cm^–2^ (Supplementary Fig. 1). Comparing our results (black and blue curves) with previous DWE reports, marked by red symbols (◊, ♦ and *), shows that the electrolytic performance of our process surpasses previous DWE reports using SRC and SRE (marked by open and solid squares, ◊ and ♦, respectively), except for the ETAC process (marked by stars, *). ETAC water electrolysis presents the lowest cell voltage, 1.5 V at a current density of 50 mA cm^–2^ (ref. ^7^), but it goes only as high as 100 mA cm^–2^, whereas our process reaches 1 A cm^–2^. Also, unlike ETAC, which is a batch process with thermal swings that require additional thermal energy to heat the hot electrolyte in the transitions from the cold stage to the hot stage^7^, our process is designed to operate continuously and isothermally (Fig. 1), avoiding this thermal loss and increasing the process productivity. As a result, the gap between the efficiency at the cell level and system level is expected to be smaller for our process. We also note that stable operation was observed in prolonged galvanostatic measurements for 5.5 h (Extended Data Fig. 6).

To assess the individual contributions of the cathodic and anodic reactions to the voltage of our process, we analysed the cathodic (HER) and anodic (bromide electro-oxidation reaction) polarization losses by means of linear sweep voltammetry (LSV) measurements in a three-electrode cell. The results are presented in Fig. 3b,c, showing the current density as a function of the potential of the Pt foil cathode and RuO_2_–TiO_2_/Ti dimensionally stable anode (DSA), respectively. These measurements were carried out in 1.5 M NaBr electrolytes containing different borate buffer concentrations (as indicated in the legend), under the same conditions as in the first operational mode of the galvanostatic measurements except for not adding Na_2_Cr_2_O_7_. One can see that the cathodic reaction (Fig. 3b) requires a much higher overpotential than the anodic counterpart (Fig. 3c). Thus, the HER presents the main polarization loss in our electrolytic process. The addition of borate buffer enhances the HER kinetics and reduces the cathodic overpotential loss substantially (Fig. 3b), with negligible effect on the anodic polarization (Fig. 3c). The beneficial effect of the borate buffer in reducing the cathodic overpotential loss may be attributed to serving as a proton source in our near-neutral NaBr electrolyte^33^ and suppressing local pH gradients at the cathode^34^.

### Catalytic process

**Extended Data Fig. 7:**
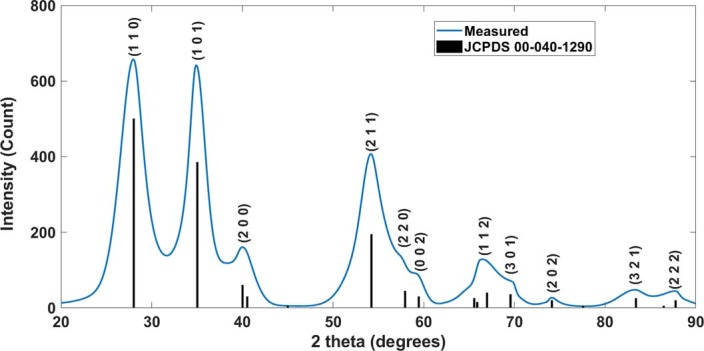
XRD of the RuO_2_ Adams catalyst. X-ray diffraction diffractogram on the RuO_2_ Adams catalyst, with reference to the JCPDS profile of the RuO_2_ rutile phase.

**Extended Data Fig. 8:**
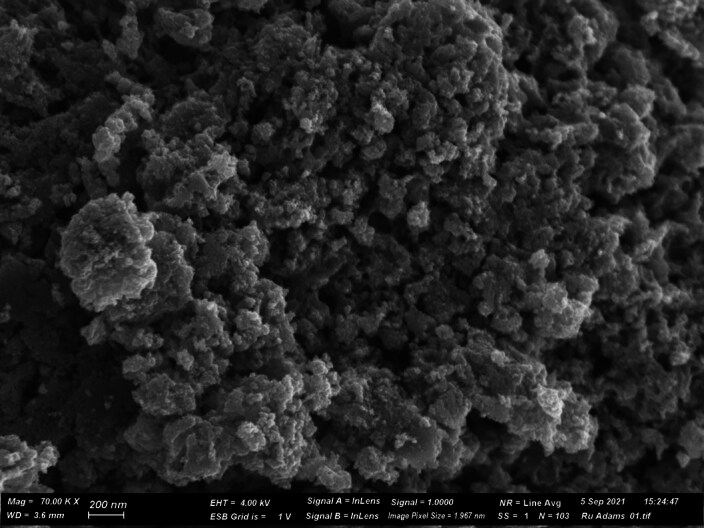
**SEM micrograph of the RuO**_**2**_ **Adams catalyst**.

**Extended Data Fig. 9:**
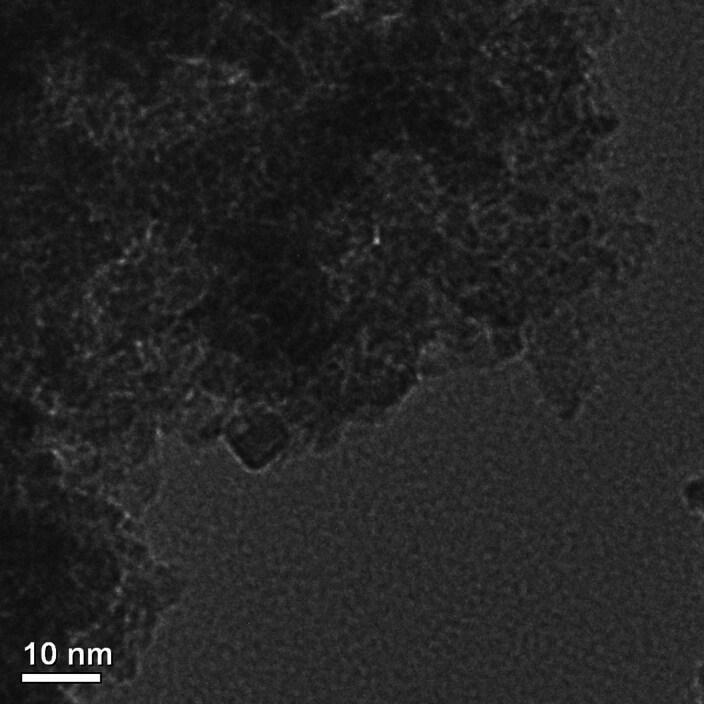
**TEM micrograph of the RuO**_**2**_ **Adams catalyst**.

The kinetics of bromate decomposition to bromide and oxygen (reaction 12) were studied using a RuO_2_ catalyst that was chosen based on previous reports^35–39^. The catalyst was synthesized using the Adams method^40^, as described in the Methods. This catalyst was composed of the rutile phase, as shown by the X-ray diffraction diffractogram presented in Extended Data Fig. 7, with a granular structure comprising submicrometre aggregates, as shown in scanning electron microscopy and transmission electron microscopy micrographs, presented in Extended Data Figs. 8 and 9, respectively, with a Brunauer–Emmett–Teller surface area of 175.3 ± 0.1 m^2^ g^–1^ (Supplementary Fig. 3b) and a bimodal pore-size distribution with 0.6–1 nm and 10–20 nm pores (Supplementary Fig. 3c). The conversion of bromate to bromide was measured by monitoring the volume of the effluent gas (oxygen) as a function of time during the catalytic decomposition of 1.5 M NaBrO_3_ aqueous solution (preheated to 60 °C) in the presence of the catalyst, using the water displacement method (Methods for details; a picture of the system is in Supplementary Fig. 4 and Supplementary Video 5). The experimental conditions of these measurements aresummarized in Table 3. Some of the catalyst was washed away by the effluent gas out of the catalytic cell. This artefact disabled precise quantitative assessment of the specific activity of the catalyst. Nevertheless, the experiments presented herein demonstrate the process functionality and performance. Future development of this process should immobilize the catalyst by embedding it in a porous support.

**Fig. 4:**
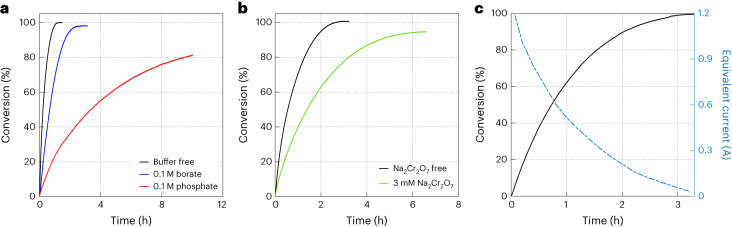
Catalytic decomposition of bromate to bromide and oxygen. **a**,**b**, Buffer effect (**a**) and Na_2_Cr_2_O_7_ effect (**b**) on the catalytic conversion of BrO_3_^–^ to Br^–^ and O_2_ (reaction 12). The electrolyte was 1.5 M NaBrO_3_ (7 ml) and the catalyst loading was 50 mg in **a** and 25 mg in **b. c**, Catalytic conversion of 1 M bromate solution obtained by bromide electrolysis (Fig. 2b). The catalyst loading was 50 mg. The solid black curve presents the fraction of conversion of bromate to bromide, and the dashed blue curve presents the equivalent electric current.

We examined the effect of borate and phosphate buffers (0.1 M) on the reaction kinetics, using ∼50 mg of the RuO_2_ Adams catalyst. Figure 4a presents the fraction of conversion of bromate to bromide as a function of time for the baseline solution (1.5 M NaBrO_3_) with and without borate and phosphate buffers. Without a buffer (black curve), full conversion was achieved in ∼1.2 h, and the initial reaction rate was 0.0362 s^–1^. Buffer addition (0.1 M) to the baseline electrolyte had an adverse effect on the bromate decomposition kinetics (Fig. 4a), which was worse for the phosphate buffer (red curve) than for the borate buffer (blue curve). For the borate buffer, the initial reaction rate was 0.0068 s^–1^, and full conversion was achieved in ∼2.5 h, whereas for the phosphate buffer the initial reaction rate dropped to 0.0041 s^–1^ and full conversion was not achieved in the time frame of this measurement (10 h). Next, the effect of the Na_2_Cr_2_O_7_ additive on the reaction kinetics was examined, using 25 mg of the RuO_2_ Adams catalyst. The results are presented in Fig. 4b. In the NaBrO_3_ electrolyte without Na_2_Cr_2_O_7_ (black curve), full conversion was achieved in ∼2.8 h and the initial reaction rate was 0.0349 s^–1^. The addition of Na_2_Cr_2_O_7_ (3 mM) reduced the initial reaction rate to 0.0168 s^–1^ and delayed the achievement of full conversion to ∼6.4 h. The catalytic deactivation in the presence of phosphate and borate buffers and Na_2_Cr_2_O_7_ additive could possibly be attributed to competitive adsorption on the surface of the catalyst that interferes with the bromate adsorption.

### Batch-to-batch process

To demonstrate the feasibility of our DWE process, we combined the electrolytic and catalytic subprocesses into a batch-to-batch process. An aliquot of 7 ml out of 20 ml of the oxidized electrolyte was taken from the best performing electrolytic test (Fig. 2b) after converting 1 M of the bromide concentration to bromate, and was transferred to the catalytic cell with 50 mg of the RuO_2_ Adams catalyst, which decomposed the bromate anions to bromide anions and oxygen. The volume of the effluent gas (oxygen) was measured (Supplementary Fig. 4) and converted to the fraction of bromate-to-bromide conversion, presented as a function of the reaction time in Fig. 4c (black solid curve). The results show complete conversion (100%) of electrolytically obtained bromate to bromide and oxygen after ∼3 h. This demonstrates a full cycle of hydrogen evolution and bromide electro-oxidation to bromate with ∼100% faradaic efficiency in the electrolytic cell, followed by complete conversion of the bromate formed in the electrolytic cell back to bromide with stoichiometric oxygen evolution in the catalytic cell.

Coupling the electrolytic and catalytic cells in a joint flow system is beyond the scope of this study, which presents a proof of concept of a new DWE process and demonstrates its basic functionality and performance. In a complete system with continuous electrolyte flow between the two cells, it is important to match the rate of bromate formation in the electrolytic cell with the rate of its conversion back to bromide in the catalytic cell. To compare these rates in our tests, the rate of oxygen evolution that was measured in the catalytic cell was converted to an equivalent electric current by assigning four electrons per O_2_ molecule, shown by the dashed blue curve in Fig. 4c. At the beginning of the reaction, the conversion rate corresponds to a high current of over 1 A. This indicates a fast catalytic reaction that would not limit the electrolytic reaction in the other cell. In future development of the continuous process, several parameters should be tuned to match the rates of the bromate formation and decomposition in the electrolytic and catalytic cells. In the electrolytic cell, the applied current, electrode size and electrolyte composition and volume should be adjusted. In the catalytic cell, the length and diameter of the catalytic column, the amount of catalyst and the porous support in which it is embedded should be properly matched with the electrolytic current and electrolyte flow rate to support a seamless continuous operation.

In summary, this work presents a new DWE process that produces hydrogen and oxygen in separate electrolytic and catalytic cells and supports continuous operation in a membraneless system. We demonstrate a high efficiency and high rate in a near-neutral electrolyte of NaBr in water, whereby bromide is electro-oxidized to bromate concurrent with hydrogen evolution in one cell, and bromate is chemically reduced to bromide in a catalytic reaction that evolves oxygen in another cell. A faradaic efficiency of 98 ± 2% was achieved in a 1.5 M NaBr electrolyte with 3.8 mM Na_2_Cr_2_O_7_ that prevents cathodic loss reactions by coating the cathode with a barrier layer that hinders the electroreduction of oxidized bromine species. Under these conditions, no oxygen evolves in the electrolytic cell, enabling safe operation without membranes. Adding a borate buffer enhances the HER and reduces the cell voltage (*IR* corrected) to 1.5 V at a current density of 5 mA cm^–2^, or 2.4 V at 1 A cm^–2^. These values correspond to electrolytic efficiency values of 97.6%_HHV_ and 61.7%_HHV_, respectively, outperforming previous reports on decoupled water splitting using electron-coupled-proton buffers^9–16^. The electrolytic efficiency of our system is slightly lower than that reported in ETAC water splitting^7^, but our process supports continuous operation without thermal swings, unlike ETAC, which is a batch process with thermal swings between cold and hot electrolytes. Another advantage of our process is operation in a near-neutral electrolyte, unlike previous reports on DWE in acid or alkaline electrolytes. We also demonstrate fast reduction of the oxidized electrolyte and oxygen evolution by a RuO_2_ catalyst without applying electricity. Further efforts to develop this breakthrough process into a competitive technology for green hydrogen production should aim at the following goals: (1) replacing the Pt cathode and RuO_2_–TiO_2_/Ti anode (DSA) and catalyst (RuO_2_ Adams) by Earth-abundant alternatives; (2) replacing the Na_2_Cr_2_O_7_ additive by non-toxic alternatives; and (3) integrating the electrolytic and catalytic subprocesses into a seamless process, and validating its long-term performance.

## Methods

### Chemicals

Double distilled water (DDW; Direct-Q3 UV, Merck) was used to prepare the aqueous solutions. Piranha solution, comprising a 2:5 mixture of concentrated hydrogen peroxide (H_2_O_2_; 30%, analytic grade, Merck) and sulfuric acid (H_2_SO_4_; 95–98%, analytical reagent grade (AR), Bio-Lab), was used for cleaning the electrochemical cells, glassware and Pt electrodes before use. Acetone (AR, Bio-Lab) and ethanol (absolute, AR, Gadot-Group) were used for cleaning the anode before use. The electrolytes were prepared using sodium bromide (NaBr; 99+%, Alfa Aesar). The pH values were adjusted with the use of sodium hydroxide (NaOH; pearls, AR, Bio-Lab) solutions. The buffer solutions were prepared with the use of boric acid (H_3_BO_3_; 99.6%, ACS grade, Acros), phosphate dipotassium phosphate (K_2_HPO_4_; ACS grade, Spectrum Chemical) and monopotassium phosphate (KH_2_PO_4_; analytic grade, Merck). Sodium dichromate dihydrate (Na_2_Cr_2_O_7_·2H_2_O; ACS grade, Merck) was used as an additive in the electrolyte. Ar gas (99.999%, Maxima) was used for purging the electrolyte in the polarization measurements. Ruthenium trichloride hydrate (RuCl_3_·H_2_O; 35–40% Ru, Acros Organics) and sodium nitrate (NaNO_3_; 99+%, ACS grade, Acros) were used for the synthesis of ruthenium dioxide powder (RuO_2_ Adams catalyst). Potassium iodide (KI; ACS reagent, Spectrum Chemical) and a standardized 0.1 N solution of sodium thiosulfate (Alfa Aesar) were used for the iodometric titration of bromate solutions. Sodium bromate (NaBrO_3_; 99.5% metal basis, Alfa Aesar) was used to prepare the solutions for the water displacement measurements.

### Electrodes

Pt foil (0.05-mm-thick foil; geometric area, 2 cm^2^; surface roughness factor, ∼3–4; 99.95%; Holland Moran) and Pt coil (wire diameter, 1 mm; coil diameter and length, 4 and 15 mm, respectively; ALS Co.) electrodes were used as cathodes in the bromide electrolysis measurements presented in Fig. 2b,c, respectively. The same Pt foil (2 cm^2^) was used as the WE in the cathodic polarization HER measurements presented in Fig. 3b. A smaller piece (0.45 cm^2^) of this Pt foil was used for the high current density measurements presented in Fig. 3a, to comply with the maximum current limitation (800 mA) of the potentiostat (BioLogic SP-150). The Pt electrodes were cleaned by dipping into piranha solution and then thoroughly rinsing in DDW before measurements. Commercial RuO_2_–TiO_2_/Ti DSAs (Ti substrate thickness, 1 mm; thickness of mixed oxide layer, ∼20–30 µm (Supplementary Fig. 2); DSA10K, De Nora) were used as anodes, keeping the geometric area close to that of the corresponding cathode. The DSAs were precleaned in a mixture of DDW/ethanol/acetone for 10 min in an ultrasound bath (MRC ultrasonic cleaner, 3 l, 120 W) and then thoroughly rinsed in DDW. A RHE (HydroFlex, Gaskatel) was used as the RE in the three-electrode measurements. It was immersed in the respective electrolyte 1 h before starting the measurements.

### RuO_2_ Adams catalyst synthesis

The RuO_2_ Adams catalyst used in the bromate reduction process (Fig. 4) was prepared following a modified Adams process^35,40^ by grinding together 2 g of RuCl_3_·H_2_O and 10 g of NaNO_3_ powders with a mortar and pestle. The resulting mixture was heated for 5 min in a box furnace (ELF Laboratory Chamber Furnace, maximum 1,100 °C, Carbolite) at 500 °C. The furnace was placed in a fume hood to safely remove toxic by-products of the reaction such as nitrogen oxide (NO/NO_2_) gases. The resulting RuO_2_ Adams catalyst was then cooled to ambient temperature and washed with DDW. The washing was repeated three times by means of centrifugation (SCEN-206 centrifuge, MRC) and decantation of the unreacted reagents in DDW. The recovered catalyst powder was then dried in air for several days.

### Faradaic efficiency measurements

The faradaic efficiency of the bromide electro-oxidation to bromate (reaction 6) was evaluated for the two operational modes under the experimental conditions described in Table 1, using a RuO_2_–TiO_2_/Ti DSA (anode) and Pt foil or coil cathode in the same compartment (Fig. 2b,d, respectively). The amount of generated bromate anions was determined at the end of each of the electrolysis tests by iodometric titration and compared with the electric charge, *I* × *t* (*I*, current; *t*, electrolysis time), that passed between the electrodes during the respective test. In the first operational mode (Fig. 2a), a double-jacketed electrochemical cell (Dr. Bob, Gamry) was used, and the electrolyte, 1.5 M NaBr without additives (experiment nos 1 and 3 in Table 1) or with 3.8 mM Na_2_Cr_2_O_7_ (experiment no. 2 in Table 1), was heated to 60 °C and stirred at 400 r.p.m. using a Teflon-coated magnetic stirrer. In the second operational mode (Fig. 2d and experiment nos 4 and 5 in Table 1), a cylindrical cell (graduated Pyrex cylinder, 20 ml, Duran) was used, and the electrolyte (1.5 M NaBr, without additives) was kept at room temperature. The initial pH of the 1.5 M NaBr electrolyte without additives was 5.8, whereas that with the Na_2_Cr_2_O_7_ additive was 7.5.

**Table 1:** Experimental conditions for faradaic efficiency measurements

Experiment no.	Figure	Faradaic efficiency (%)	Na_2_Cr_2_O_7_	Cell type	Cathode	Cathode coating	Temp. (°C)	Stirring (r.p.m.)	pH, initial	pH, final
1		10 ± 1	-	Double-jacketed cell	Pt foil	-	60	400	5.8	9.2
2	2a,b	98 ± 2	3.8 mM			In situ^a^			7.5	7.9
3		80 ± 2	-			Ex situ^b^			5.8	9.5
4	2c–e	72 ± 2	-	Cylindrical cell	Pt coil	-	r.t.	-	5.8	8.3
5		13 ± 1	-					400	5.8	10.5

Electrolysis current, 600 mA; electrolysis duration, 5.36 h; 20 ml of 1.5 M NaBr electrolyte (without buffer); r.t., room temperature.

^a^In situ coating by electroreduction of Cr_2_O_7_^2–^ anions and cathodic deposition during electrolysis.

^b^Using a precoated cathode from prior electrolysis tests.

### Long-term stability

Faradaic efficiency measurements were carried out during five consecutive days with 119 h of continuous electrolysis at a current of 300 mA (current density of 150 mA cm^–2^). A large (500 ml) cylindrical cell with a Pt foil cathode and DSA (anode) was filled with 300 ml of the electrolyte (initial concentration, 1.5 M NaBr, 0.3 M borate buffer and 3.8 mM Na_2_Cr_2_O_7_; photograph in Extended Data Fig. 1). The cell was heated to 60 °C and stirred at 400 r.p.m. To reduce evaporation, the cap was sealed to the cell with a Parafilm laboratory film. The bromide and bromate concentrations were measured by sampling the electrolyte approximately every 24 h during the test, and analysing the sample composition by iodometric titration and ion chromatography. The results are presented in Extended Data Figs. 2 and 3, respectively. The pH was measured each day, and the results were in the range of 8.0 to 8.6.

### Iodometric titration

Iodometric titration^41,42^ was used to determine the concentration of bromate anions (${{\rm{BrO}}}_{3}^{-}$) in the electrolyte after bromide electrolysis tests. The bromate anions were reduced to bromide anions (Br^–^) in the presence of an excessive amount of iodide anions in acidic medium: ${\rm{Br}}{{\rm{O}}}_{3}^{-}+6{{\rm{I}}}^{-}+3{{\rm{H}}}_{2}{\rm{S}}{{\rm{O}}}_{4}\to 3{{\rm{I}}}_{2}+{{\rm{Br}}}^{-}+3{\rm{S}}{{\rm{O}}}_{4}^{2-}+3{{\rm{H}}}_{2}{\rm{O}}$. The iodine (I_2_) molecules that evolved were titrated by standardized thiosulfate solution: 2Na_2_S_2_O_3_ + I_2_ → 2NaI + Na_2_S_4_O_6_. To this end, a solution of 3.8 g KI in 40 ml DDW was added to an aliquot of 400 µl (volume of aliquot, *V*_aliquot_) that was collected from the electrolytic cell (volume of cell, *V*_electrolyte_ = 20 ml) at the end of the electrolysis experiment, followed by the addition 0.6 ml of concentrated sulfuric acid, H_2_SO_4_. After dilution by DDW to a volume of 50 ml, the resultant dark-violet solution (Supplementary Fig. 5, left) was gradually titrated by adding a standardized 0.1 N solution of sodium thiosulfate, Na_2_S_2_O_3_ (concentration of thiosulfate anion, ${C}_{{{\rm{S}}}_{2}{{\rm{O}}}_{3}^{2-}}=0.1 \ {\rm{M}}$) until reaching a transparent solution (Supplementary Video 3 and Supplementary Fig. 5, right). The volume of the thiosulfate solution that was added to this point, ${V}_{{{\rm{S}}}_{2}{{\rm{O}}}_{3}^{2-}}$, was used to calculate the amount of bromate ions that was generated by the electrolysis: ${n}_{{\rm{Br}}{{\rm{O}}}_{3}^{-}}=\frac{1}{6}\frac{{V}_{{\rm{electrolyte}}}}{{V}_{{\rm{aliquot}}}}{V}_{{{\rm{S}}}_{2}{{\rm{O}}}_{3}^{2-}}{C}_{{{\rm{S}}}_{2}{{\rm{O}}}_{3}^{2-}}$.

### Ion chromatography

Ion chromatography was used to measure the concentration of bromide and bromate anions in the sampled solutions during the long-term stability measurement (Extended Data Figs. 2 and 3, respectively) using a Metrohm 881 Compact IC pro chromatograph equipped with a Shodex IC-SI-52 4E analytical column. Two 100 μl duplicate samples were collected approximately every 24 h from the beginning of the test and diluted by DDW to prepare 4 ml samples that were analysed by ion chromatography using bromate and bromide standards from Sigma-Aldrich.

### Electrolytic efficiency

The electrolytic efficiency of the bromide electrolysis tests was evaluated from the steady-state current–voltage results presented in Fig. 3a. To this end, galvanostatic measurements were carried out at different currents in a two-electrode cell configuration (Fig. 2a) using a BioLogic SP-150 potentiostat with a Pt foil cathode and a DSA10K anode. The electrolyte was 1.5 M NaBr with sodium dichromate additive (3.8 mM), without a buffer or with 0.7 M borate buffer (black and blue ● symbols in Fig. 3a, respectively). The electrolyte was heated to 60 °C in a double-jacketed electrochemical cell (Dr. Bob, Gamry) and stirred at 400 r.p.m. Th experimental conditions are summarized in Table 2. Current density values from 5 to 1,000 mA cm^–2^ were applied in ascending order, holding for 5 min at a time, while monitoring the applied voltage. The voltage values were averaged to obtain the mean value and standard deviation at each current density and corrected for the ohmic (*IR*) drop. The resistance *R* was measured by electrochemical impedance spectroscopy using a BioLogic SP-150 potentiostat and was determined as the high-frequency intercept with the real axis in the Nyquist plot. The electrochemical impedance spectroscopy measurements were conducted in galvanostatic mode at the same currents as the current–voltage measurements, with an oscillation amplitude of 5% of the mean current and oscillation frequency from 200 kHz to 100 mHz. The *IR*-corrected mean voltage values, *V*_cell_, were used to calculate the electrolytic efficiency based on the higher heating value (HHV) of hydrogen: *η* = (1.48/*V*_cell_) × 100 (in units of percent HHV).

**Table 2:** Experimental conditions for electrolytic efficiency and cathodic and anodic polarization measurements

Figure	Method	Electrodes	Electrolyte	Buffer	Stirring
3a	Galvanostatic two-electrode cell	DSA (anode) Pt foil cathode	1.5 M NaBr 3.8 mM Na_2_Cr_2_O_7_	-	400 r.p.m.
				0.7 M borate	
3b	LSV three-electrode cell	Pt foil WE Pt wire CE RHE RE	1.5 M NaBr	-	-
				0.1 M borate	
				0.4 M borate	
				0.7 M borate	
3c	LSV three-electrode cell	DSA WE Pt coil CE RHE RE	1.5 M NaBr	0.1 M borate	-
				0.4 M borate	
				0.7 M borate	

All the measurements were carried out in a heated electrolyte (60 °C). The initial pH was 8. CE, counter electrode; RE, reference electrode; WE, working electrode; RHE, reversible hydrogen electrode.

### Cathodic polarization measurements

The cathodic polarization curves presented in Fig. 3b were measured by LSV in a three-electrode water-jacketed electrochemical cell (Dr. Bob, Gamry) using Pt foil (2 cm^2^) as the WE and a potentiostat (BioLogic SP-150) at a scan rate 1 mV s^–1^. A Pt wire (diameter, 1 mm; Gamry) placed in a separate fritted glass compartment was used as the CE, where bromide electro-oxidation took place. A RHE was used as the RE. The RE and WE were placed in the same compartment. The series resistance *R* was measured by a current interruption method before each measurement. Measurements were carried out in Ar-purged 1.5 M NaBr electrolytes containing different additives at 60 °C. The basicity of the electrolyte was adjusted to pH 8 using a 5 M NaOH solution, measured by an Oakton series 500 pH meter. The experimental conditions are summarized in Table 2. In short experiments that correspond to several polarization curves, the addition of sodium dichromate to the electrolyte was found to have a negligible effect on the polarization curves; therefore it was not used in the cathodic polarization measurements. No stirring was applied in these measurements to minimize interference by back reactions of the products of bromide electro-oxidation at the CE.

### Anodic polarization measurements

The anodic polarization curves presented in Fig. 3c were measured similarly to the cathodic polarization measurements, with the following exceptions: first, a DSA10K anode (2 cm^2^) was used as the WE; and second, the WE and CE (Pt coil) were placed in the same compartment, so that the hydroxide anions (OH^–^) formed at the CE would not be restricted from reacting with Br_2_ formed at the WE in the bulk solution. The experimental conditions are summarized in Table 2.

### Bromate catalytic decomposition

A water displacement technique^43–45^ was used to monitor the kinetics of the bromate catalytic decomposition (reaction 12) by the RuO_2_ Adams catalyst (Fig. 4). The method is based on weighing the amount of water displaced by the oxygen released in the reaction. The experimental set-up, presented in Supplementary Fig. 4, comprises a gas-tight glass reactor placed in a water bath on top of a heated plate and a water column. The outlet of the reactor is connected to the inlet of a water column, whereas the outlet of the column is directed to a beaker that is placed on a digital balance (BB-1550, MRC) that monitors the weight change as a function of time during the decomposition reaction. Before the start of the experiment, the silicone tube connecting the reactor and the water column is closed by a metal pinch clamp. To start the measurement, a known volume (*V*_sol_ = 7 ml) of NaBrO_3_ solution with a known concentration of bromate anions (${C}_{{{\rm{BrO}}}_{3}^{-}}=1.5 \ {\rm{M}}$) is added to the reactor, which contains a measured mass of the RuO_2_ Adams catalyst (${m}_{{{\rm{RuO}}}_{2}}$). Then, the reactor is sealed, and the pinch clamp is opened. The oxygen gas that evolves in the reactor flows to the water column through the tube and displaces the water from the column to the beaker, and the mass of the displaced water (*m*_water_) is constantly measured (Supplementary Video 5). The measured mass (in kilograms) is nearly equal to the oxygen volume (${V}_{{{\rm{O}}}_{2}}$, in litres) since the density of water is 0.998 kg l^–1^ at standard temperature and pressure. The oxygen volume, ${V}_{{{\rm{O}}}_{2}}$, is converted to number of moles of oxygen, ${n}_{{{\rm{O}}}_{2}}$, by the ideal gas law (*PV* = *nRT*; *P*, pressure of gas; *V*, volume of gas; *n*, moles of gas; *R*, ideal gas constant; *T*, temperature of gas). Taking a ratio of 2:3 between the bromate anions converted to bromide and the generated O_2_ molecules (reaction 12), the conversion fraction is ${2n_{{\mathrm{O}}_2}}/{3V_{{\mathrm{sol}}}}{{C}_{{{\rm{BrO}}}_{3}^{-}}}$, presented in Fig. 4 as a function of the reaction time (*t*). The slope $s={{\mathrm{d}}n_{{\mathrm{O}}_2}}/{{\mathrm{d}}t}$ yields a reaction rate that can be converted to an equivalent electric current *I* = 4 × *F* × *s* (presented in Fig. 4c, blue curve), where *F* is Faraday’s constant and 4 is the number of electrons needed to generate an O_2_ molecule. The conversion values were verified independently by end-of-experiment iodometric titrations, confirming the full decomposition of bromate to bromide with close to 100% oxygen yield at the end of the experiments. The water displacement technique was applied for a series of experiments with different catalyst-to-solution volume ratios and with different electrolyte additives including dichromate, phosphate buffer and borate buffer, as summarized in Table 3.

**Table 3:** Experimental conditions for catalytic decomposition experiments

Figure	Initial rate (s^–1^)	RuO_2_ Adams mass (mg)	Additives
**–**	0.0310	102.8	No
4a	0.0362	51.2	
4b	0.0349	25.5	
4a	0.0041	51.6	0.1 M phosphate buffer
4a	0.0068	55.0	0.1 M borate buffer
4b	0.0168	25.4	3 mM Cr_2_O_7_^2−^

All the measurements were carried out in a heated and stirred 1.5 M NaBrO_3_ electrolyte (60 °C, 400 r.p.m.). The initial pH was 8.

## Online content

Any methods, additional references, Nature Portfolio reporting summaries, source data, extended data, supplementary information, acknowledgements, peer review information; details of author contributions and competing interests; and statements of data and code availability are available at 10.1038/s41563-023-01767-y.

### Supplementary information

Supplementary Information Supplementary Figs. 1–5, Table 1 and Discussion.

Supplementary Video 1 Bromide electrolysis without steering. Hydrogen bubbles evolve at the cathode on the left and accumulate at the top part of the cell. The reddish oxidized electrolyte sinks down from the anode (at the centre of the cell) and accumulates at the bottom of the cell.

Supplementary Video 2 Subsequent steering following bromide electrolysis without steering. The phase-separated solution that evolved during the bromide electrolysis test without steering (Supplementary Video 1) is mixed and becomes homogeneous during subsequent steering.

Supplementary Video 3 Iodometric titration to determine the concentration of bromate anions in the electrolysed solution, measured by adding thiosulfate solution to titrate the red iodine (I_2_) molecules, which evolved by reducing the bromate anions formed in the electrolysis test with potassium iodide (KI), to a colourless solution.

Supplementary Video 4a Bromide electrolysis in a Hoffman apparatus with separated cathodic (left) and anodic (right) compartments, showing hydrogen evolution at the cathode and no gas evolution at the anode. This video clip was taken at the beginning of the test, before the evolved hydrogen accumulated in the cathodic cell.

Supplementary Video 4b Bromide electrolysis in a Hoffman apparatus with separated cathodic (left) and anodic (right) compartments, showing hydrogen evolution at the cathode and no gas evolution at the anode. This video clip was taken during the middle of the test, showing excessive hydrogen evolution at the cathode and accumulation at the top part of the cathodic compartment. The anode oxidizes the yellowish electrolyte to a red oxidized solution, without any gas (that is, oxygen) evolution.

Supplementary Video 4c Bromide electrolysis in a Hoffman apparatus with separated cathodic (left) and anodic (right) compartments. This video clip was taken at the end of the test, showing the amount of hydrogen gas that accumulated at the top part of the cathodic compartment, and no gas accumulation in the anodic compartment, proving that no oxygen evolved during the test.

Supplementary Video 5 The catalytic conversion of bromate to bromide with oxygen evolution using a RuO_2_ Adams catalyst. The amount of oxygen evolved during the test is measured by water displacement and weighing the amount of water with a scale on the right side. Note the displacement of a small amount of the catalyst by the effluent oxygen gas, browning the silicone tube that connects the catalytic cell and the water displacement column.

## Figures and Tables

**Extended Data Fig. 10:**
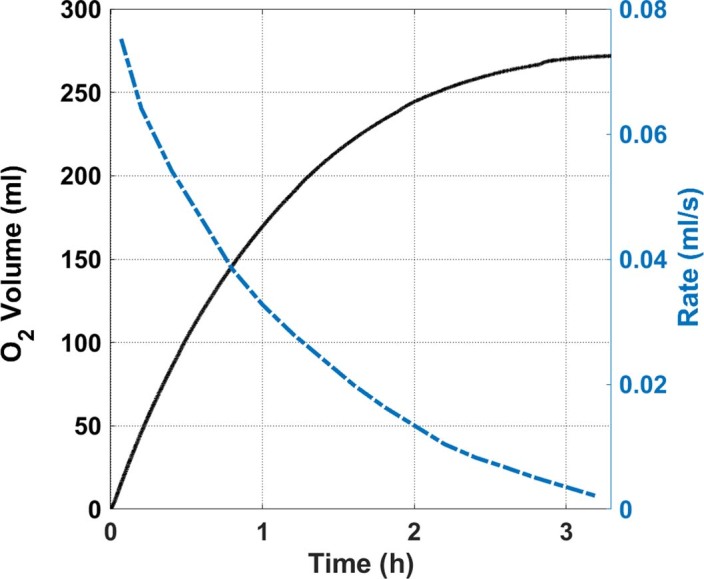
Catalytic conversion of 1 M bromate solution obtained by bromide electrolysis. The solid black curve presents the evolved volume of oxygen due to bromate decomposition, and the dashed blue curve presents the oxygen evolution rate.

## Data Availability

The authors declare that the data supporting the findings of this study are available within the paper and its Supplementary Information files.
