# Supplementary material for: Electrochemical and chemical cycle for high-efficiency decoupled water splitting in a near-neutral electrolyte
Source: Nat Mater. 2024 Jan 9;23(3):398–405. doi: 10.1038/s41563-023-01767-y (PMC10917665; doi:10.1038/s41563-023-01767-y)
Supplement: Supplementary file 1 — Supplementary Figs. 1–5, Table 1 and Discussion. [file 41563_2023_1767_MOESM1_ESM.pdf]

nature materials

Article

https://doi.org/10.1038/s41563-023-01767-y

Electrochemical and chemical cycle for high-efficiency decoupled water splitting in a near-neutral electrolyte

In the format provided by the authors and unedited

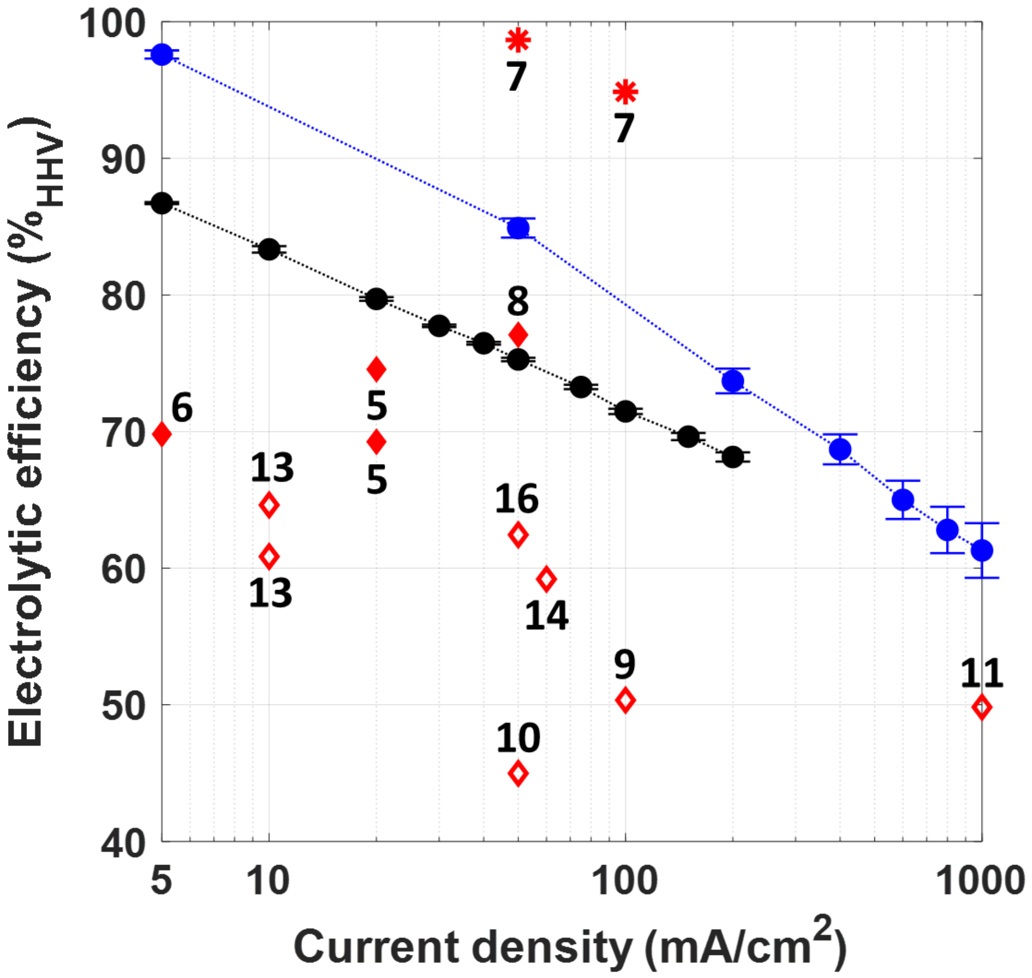

Figure S1. **Electrolytic efficiency.** The electrolytic efficiency, 1.48/(V − IR)×100%$_{HHV}$, at different current density values, calculated from the results presented in **Figure 3a**, using the same color code and reference labeling.

Table S1. Electrolytic efficiency. The results of the electrolytic efficiency measurements presented in **Figure 3a** without **(a)** and with **(b)** (0.7M) borate buffer. The electrode area in **(a)** is larger than in **(b)**, 2 and 0.45 cm^2, respectively. As a result, the series resistance in **(a)** is smaller than in **(b)**. The electrode distance was larger in **(a)** than in **(b)**, therefore the respective series resistance values do not scale according to the electrode area ratio (1 : 4.4). The voltage values and error bars present the mean values and standard deviation of the galvanostatic measurements presented in **Extended Data Figure 2**.

(a)

**Voltage (V)**	1.734	1.831	1.967	2.069	2.158	2.242	2.436	2.625	2.966	3.292
**Current (mA)**	10	20	40	60	80	100	150	200	300	400
**Resistance (Ω)**	2.7	2.8	2.8	2.8	2.8	2.8	2.8	2.8	2.8	2.8
**Current density (mA/cm^2)**	5	10	20	30	40	50	75	100	150	200
**Voltage IR corrected (V)**	1.707	1.776	1.857	1.904	1.935	1.966	2.020	2.071	2.126	2.172
**Error bars (V)**	0.001	0.005	0.003	0.002	0.003	0.003	0.004	0.006	0.008	0.011

(b)

**Voltage (V)**	1.525	1.831	2.361	2.878	3.399	3.895	4.376
**Current (mA)**	2.25	22.5	90	180	270	360	450
**Resistance (Ω)**	4.1	3.9	3.9	4.0	4.2	4.3	4.4
**Current density (mA/cm^2)**	5	50	200	400	600	800	1000
**Voltage IR corrected (V)**	1.52	1.74	2.01	2.15	2.28	2.36	2.41
**Error bars (V)**	0.01	0.01	0.03	0.04	0.05	0.063	0.08

DSA anode and RuO_2 Adams catalyst characterizations

X-ray diffraction. Crystallographic phase analysis of the RuO_2 Adams catalyst was carried out by powder X-ray diffraction (XRD). The diffractograms were collected with a Smartlab 9 kW Rigaku diffractometer using Cu $K\alpha_1$ radiation (λ = 0.15406 nm) with a tube current of 150 mA and voltage of 45 kV, in the parallel beam geometry, scanning $\theta/2\theta$ at the rate of 1°/min in 0.01° steps from 20° to 90°. The crystallographic phases were identified using the Match software (version 311.3.191) to fit the peaks in the X-ray diffractograms with Bragg reflections of the respective phases, for which we used the International Centre for Diffraction Data (ICDD) database and the Inorganic Crystal Structure (ICSD) database (PDXL2 software, version 2.8.1.1). The X-ray diffractogram of the prepared RuO_2 Adams catalyst is presented in **Extended Data Figure 4a**.

Scanning electron microscopy. The surface morphology of the RuO_2 Adams catalyst and DSA anode was examined by high-resolution scanning electron microscopy (HRSEM), using a Zeiss Ultra Plus field-emission microscope operated at an accelerating voltage of 4 kV. Selected HRSEM micrographs of the RuO_2 Adams catalyst are presented in **Extended Data Figure 4b**, and a selected SEM micrograph of the DSA anode is presented in **Figure S2**.

Transmission electron microscopy. The morphology of the RuO_2 Adams catalyst was examined by transmission electron microscopy (TEM), using a FEI Tecnai T20 LaB6 field-emission gun (FEG) microscope operated at an accelerating voltage of 200 kV. The catalyst powder (2 mg) was dispersed in isopropanol (10 ml) and placed in an ultrasound bath for 10 min. The resulting dispersion was drop-casted onto a holey carbon coated Cu grid (300 mesh, 3 mm diameter, SPI Supplies) and left to dry at ambient temperature in a desiccator. A TEM micrograph of the RuO_2 Adams catalyst is presented in **Extended Data Figure 4c**.

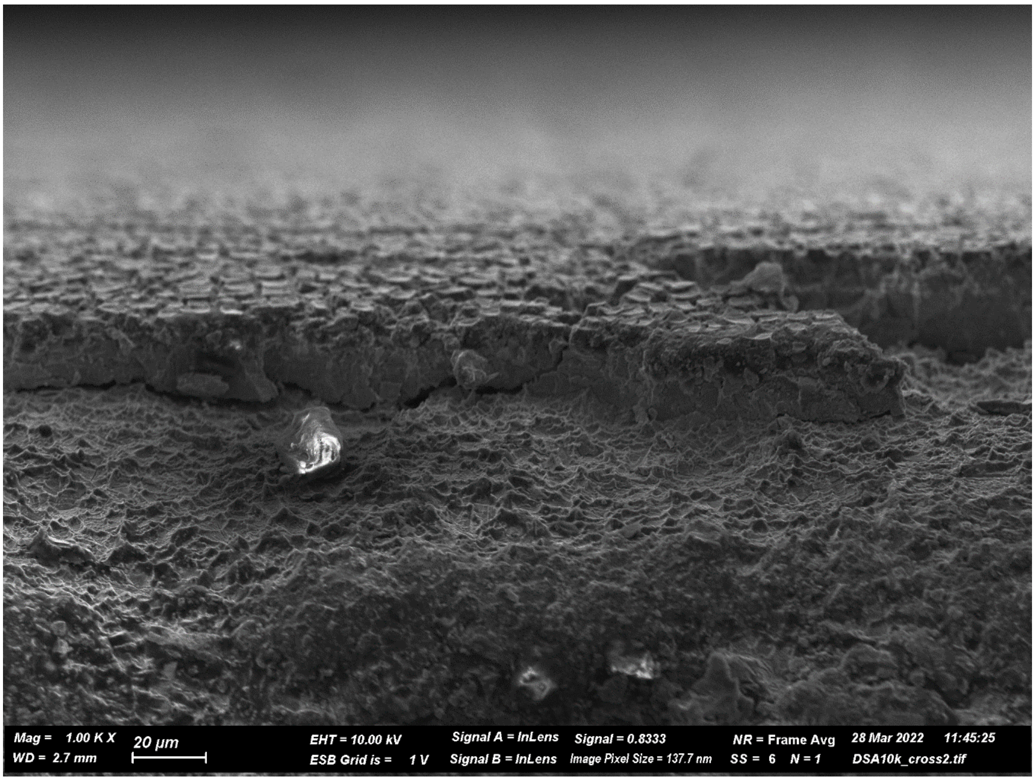

Figure S2. Tilted SEM micrograph of the DSA anode.

Nitrogen physisorption. N_2 physisorption was used to determine the surface area of the RuO_2 Adams catalyst, using a 3Flex adsorption analyzer (Micromeritics, USA). Before measurement, the sample was weighed with an analytical balance, loaded into the sample tube, and degassed in a Smart VacPrep gas adsorption sample preparation device (Micromeritics, USA) for 24 h at 120°C (temperature ramp rate of 10°C/min) to completely remove all the moisture. Final degassing was performed on the 3Flex analyzer for 3 h at 120°C (temperature ramp rate of 10°C/min). After degassing and backfilling with N_2 (99.999%, Maxima, Israel), the samples were analyzed at liquid N_2 temperature (77K) in a pressure of 101.3 kPa (p_0), and N_2 adsorption/desorption isotherms were obtained in the relative pressure (p/p_0) range of 0.001 to 1, with equilibration intervals of 10 s. The resulting N_2 physisorption isotherm is presented in **Figure S3a**. Based on the adsorption branch, the BET (Brunauer Emmet Teller) model was used to calculate the specific surface area (**Figure S3b**) and pores size values, and DFT (Density Function Theory) model was used to analyze the pore size distribution (**Figure S3c**).

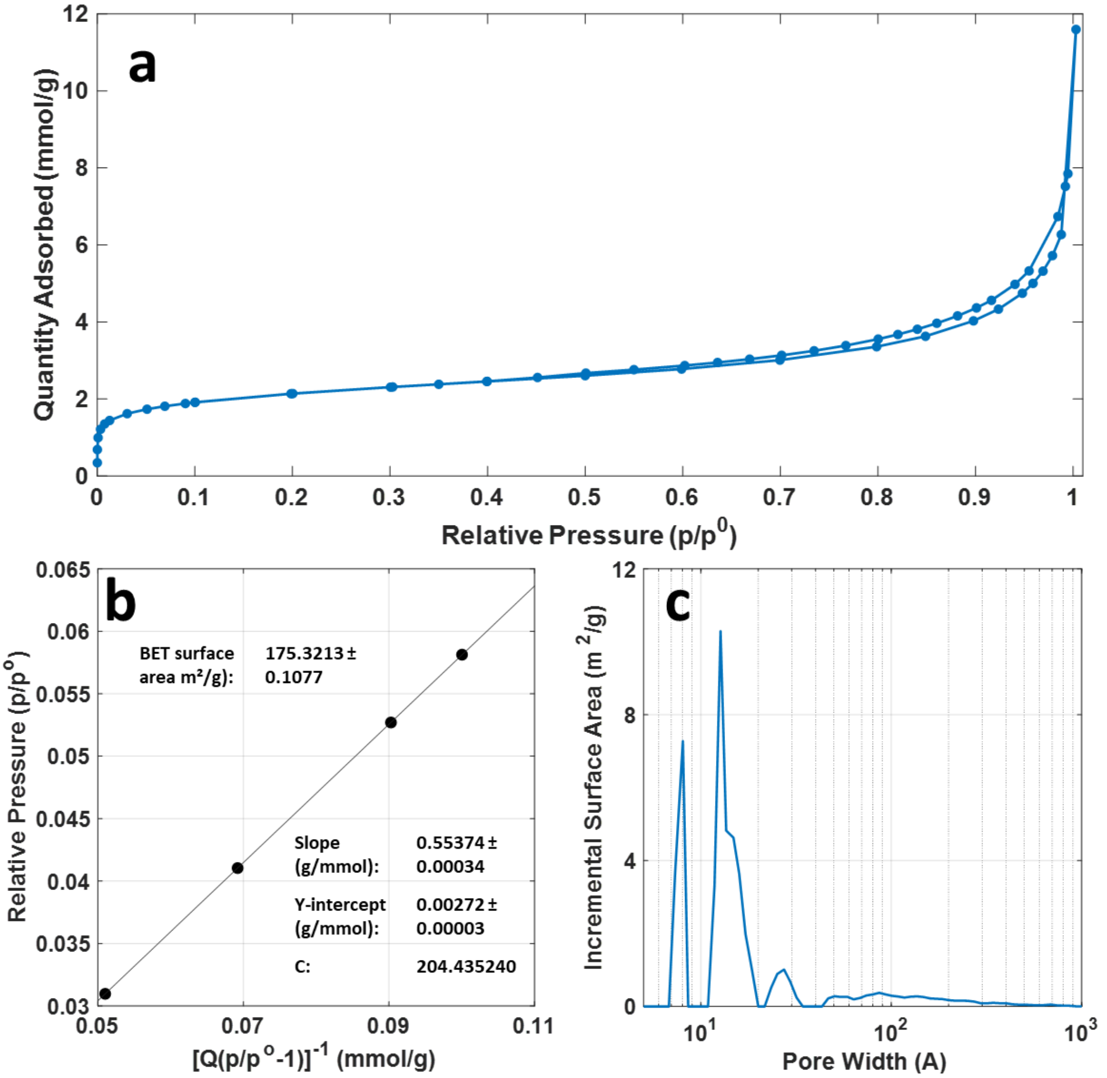

Figure S3. **(a)** N_2 physisorption isotherm for the RuO_2 Adams catalyst; **(b)** surface area by BET method; **(c)** pore size distribution.

Water displacement

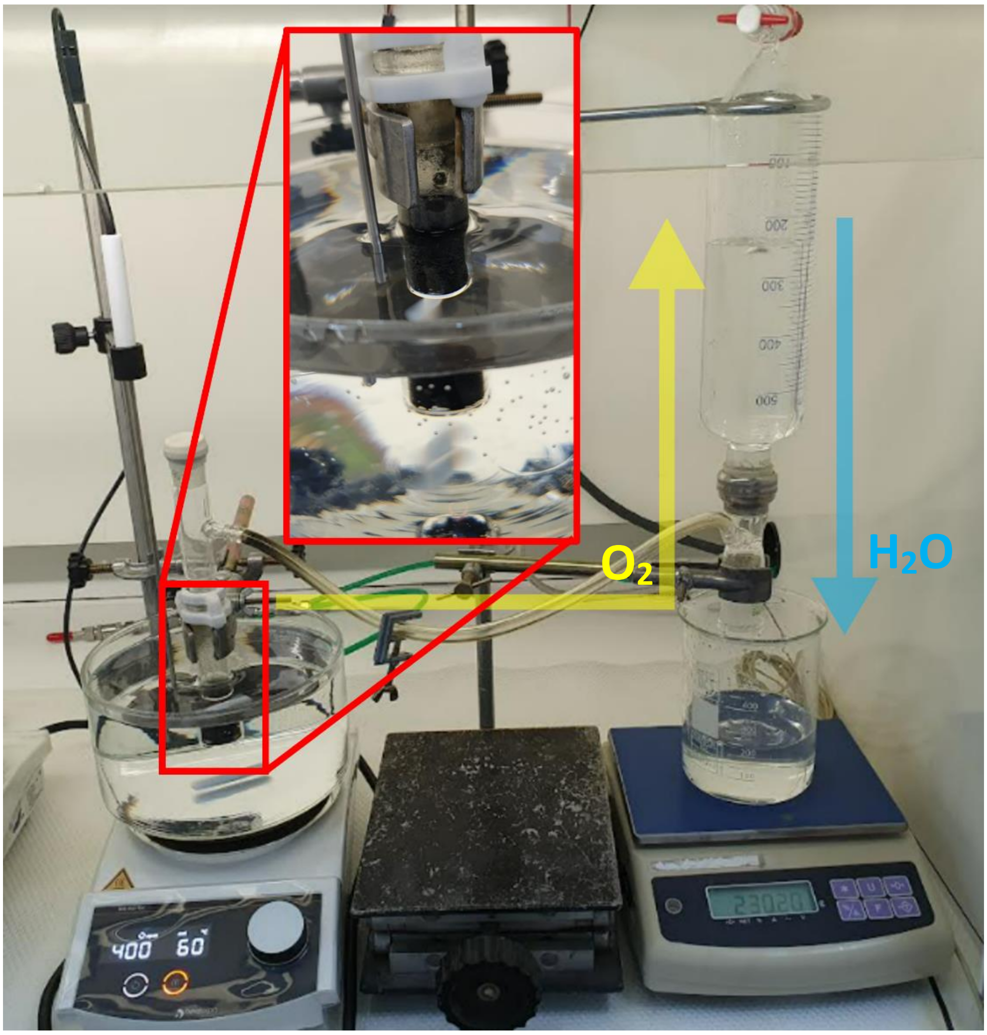

Figure S4. Water displacement experimental setup.

Iodometric titration

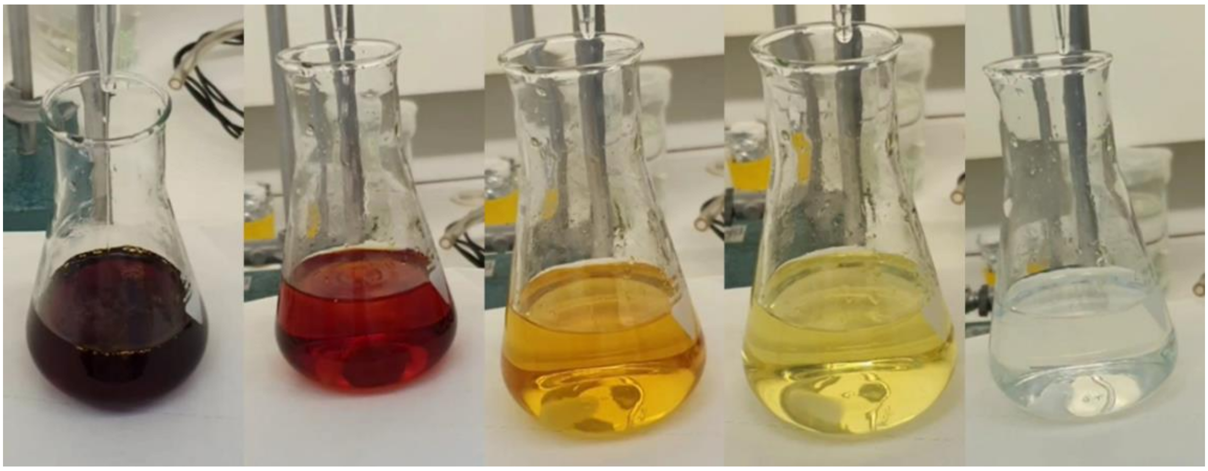

Figure S5. Iodometric titration progress from left to right. The color is attributed to I_2 that was initially high in concentration (dark red, left photograph) and upon titration it was reduced until no I_2 was left, and the solution turned colorless (right photograph).
